# Repression of *Staphylococcus aureus* and *Escherichia coli* by *Lactiplantibacillus plantarum* Strain AG10 in *Drosophila melanogaster* In Vivo Model

**DOI:** 10.3390/microorganisms11051297

**Published:** 2023-05-16

**Authors:** Elizaveta Gavrilova, Victoria Kostenko, Iva Zadorina, Dilyara Khusnutdinova, Dina Yarullina, Asya Ezhkova, Mikhail Bogachev, Airat Kayumov, Elena Nikitina

**Affiliations:** 1 Institute of Fundamental Medicine and Biology, Kazan Federal University, 420008 Kazan, Russia; 2 Department of Physiology and Patophysiology, Kazan State Academy of Veterinary Medicine Named after N.E. Bauman, 420029 Kazan, Russia; 3 Biomedical Engineering Research Centre, St. Petersburg Electrotechnical University, 197022 St. Petersburg, Russia; 4 Department of Meat and Milk Technology, Kazan National Research Technological University, 420015 Kazan, Russia

**Keywords:** antimicrobial peptides, *Drosophila melanogaster*, in vivo antagonistic activity, lactic acid bacteria

## Abstract

Probiotic bacteria exhibiting antagonistic activities against pathogenic bacteria are widely considered as potential options for the prevention and treatment of various infectious diseases and represent potential substitutes of antibiotics. Here we show that the *L. plantarum* AG10 strain represses the growth of *Staphylococcus aureus* and *Escherichia coli* in vitro and diminishes their negative effects in vivo in a *Drosophila melanogaster* model of survival on embryonic (larvae) and pupa stages. In an agar drop diffusion test, *L. plantarum* AG10 exhibited antagonistic properties against *Escherichia coli*, *Staphylococcus aureus*, *Serratia marcescens* and *Pseudomonas aeruginosa*, and repressed the growth of *E. coli* and *S. aureus* during milk fermentation. In a *Drosophila melanogaster* model, *L. plantarum* AG10 alone did not provide any significant effect, either during the embryonic stage or during further development of the flies. Despite this, it was able to restore the viability of groups infected with either *E. coli* and *S. aureus*, almost to the level of non-treated control at all stages of development (larvae, pupa and adult). Moreover, in the presence of *L. plantarum* AG10, pathogens-induced mutation rates and recombination events reduced 1.5–2-fold. The genome of *L. plantarum* AG10 was sequenced and deposited at NCBI under the accession number PRJNA953814 and consists of annotated genome and raw sequence data. It consists of 109 contigs and is 3,479,919 bp in length with a GC content of 44.5%. The analysis of the genome has revealed considerably few putative virulence factors and three genes responsible for the biosynthesis of putative antimicrobial peptides, with one of them exhibiting a high probability of antimicrobial properties. Taken together, these data allow the suggestion that the *L. plantarum* AG10 strain is promising for use in both dairy production and probiotics as a preservative from foodborne infections.

## 1. Introduction

Since the discovery of penicillin by Alexander Fleming in 1929, various antimicrobials have been designed and translated into clinical practice. Along with their active use in human and veterinary medicine for infection treatment, they have been intensively used in livestock and poultry as animal growth enhancers and diseases prevention tools since at least the 1940s [1]. As a result, antibiotics continuously accumulate in soils and water, thereby causing either resistance development or the acquisition of resistance genes by bacteria [2,3]. The growing spread of bacterial resistance to antimicrobials threatens the health of both humans and animals; this is emerging as a significant global health challenge. Despite legal restrictions on antibiotic use in some countries, their consumption is expected to increase by more than 60 percent by 2030 [4,5,6], challenging the development of their environmentally friendly and natural alternatives.

Various food supplements have been suggested as substitutes for antibiotics. In some studies, organic acids (formic, acetic or propionic) were added to drinking water or animal feed, leading to decreased number of bacteria (*Salmonella* and *Campylobacter*) in the intestines of poultry [7,8,9]. To suppress bacterial growth (such as *Salmonella*, *Clostridium* and *Escherichia coli*), various plant extracts and essential oils (for example, thyme, cinnamon, black pepper, and many others) have also been investigated [10,11,12,13]. The composition of these plant extracts may include carotenoids, phenols, alkaloids, terpenes, peptides and many others [14,15,16]. Antimicrobial peptides can be isolated not only from plants, but also from completely different origins: animals, insects or created using genetic engineering methods [17].

Probiotic bacteria with antagonistic activities against pathogens are often considered as promising substitutes to antibiotics [18,19,20]. The Food and Agriculture Organization of the United Nations (FAO) and the World Health Organization (WHO) defined probiotics as “live microorganisms which when administered in adequate amounts confer a health benefit on the host” [21]. The development of probiotic drugs and attendant scientific and clinical research continues to develop at pace [22]. In animal husbandry, poultry and aquaculture, probiotics are used as growth stimulants as well as for the prevention and treatment of diseases [23,24]. Among several prominent examples, *Lactococci*, *Lactobacilli*, *Enterococci* and *Bacilli* are commonly used [25,26].

Although the intestinal microbiota represents a huge complex community of various bacteria, many species of the genera *Lactobacillus* and *Bifidobacterium* have been shown to offer protection against enteric infections. Several species of the genus *Lactococci* are intentionally introduced in the food chain, being involved in a range of food and feed fermentations, and are applied as probiotics in humans and animals [27,28]. The use of viable spores of *Bacillus* as a probiotic supplement raised a number of questions, including their safety; several *Bacillus* species used as animal feed supplements, probiotics, plant protection products or seed coating agents are also known as agents of food poisoning. However, they are widely used in animal husbandry, in particular as a probiotic supplement for the swine livestock [29,30].

Lactic acid bacteria (LAB) typically exhibit strong antagonism against other bacteria by using various tools, including synthesis of organic acids (mainly lactic acid) and acidification of the broth [31], hydrogen peroxide production [32] and secretion of antimicrobial peptides as well as bacteriocins [33,34]. For example, in vaginal secretions, probiotic *Lactobacilli* exhibit an acidifying ability, synthesize hydrogen peroxide and form co-aggregates with pathogenic strains of *Streptococcus agalactiae* [35]. The probiotic strain *L. diolivorans* 1Z contributed to animal survival after infection by *Salmonella enterica* (*Typhimurium* serotype) due to colonization resistance and immunomodulation of the host intestine [36]. Oral probiotics have been evaluated for their possible application in the prevention and treatment of caries in an in vivo rat model, indicating that lactobacillus strains exhibit strong colonization resistance and are capable of destroying biofilms of caries-inducing *Streptococcus mutans* [37]. Moreover, probiotic *Lactobacilli* can stimulate the growth and maturation of animals, improving both the innate and the acquired immune mechanisms of the host [38,39]. They produce lactic acid as the major metabolic end product of carbohydrate fermentation while also supporting food digestion via production of hydrogen peroxide and other substances, thus creating an unfavorable acidic environment for harmful or pathogenic organisms [40,41,42]. Thus, direct activity of *Lactobacilli* on other microorganisms prevents the development of infections and serves as an effective tool to control and restore the microbial equilibrium in the gut [43,44]. Furthermore, various bacterial genera possess genotoxins, for example, the cytolethal distending toxin (CDT), which causes DNA double-strand breaks (DSBs) [45]. Others alter the host DNA damage response, potentially resulting in mutations and cancer (reviewed in [46]). A deleterious action on host DNA integrity has been described for Gram-negative bacteria such as *Helicobacter* sp., *Chlamydia* sp., *Salmonella* sp. or *Escherichia coli*, demonstrating that these mechanisms may lead to genomic alterations and transformations associated with cancer development [46]. *S. aureus* also was shown to trigger ROS-mediated DNA damage, thus affecting their genomic integrity [47]. Thus, the decrease of pathogenic bacteria in the gut by their substitution by probiotic bacteria would explain, at least partially, their antimutagenic activity (reviewed in [48,49]).

In previous studies, several strains of lactic acid bacteria with probiotic properties were isolated from clover silage [50,51,52]. Among them, *L. plantarum* AG10 demonstrated the most promising probiotic features, as well as antimicrobial activity against pathogens in both liquid medium and biofilm. Furthermore, during milk fermented by *L. plantarum* AG10, the dry matter content and product density were slightly higher than in the milk fermented by conventional industrial strains [53]. At the same time, it produces less lactic acid during milk fermentation and, as a result, has a lower acidification rate. On the other hand, the amount of EPS in fermented milk *L. plantarum* AG10 is higher when compared to a control industrial strain [52]. As a consequence of the larger production of expanded polystyrene, fat-free samples obtained from *L. plantarum* AG10 fermented milk demonstrated significantly higher viscosity, product adhesion and higher resistance to destruction [53].

*Drosophila melanogaster* is a common and convenient model organism widely used to study the effects of the intestinal microbiota and the effects of probiotics on the host organism. Flies have several features that make them suitable model organisms [54]. Their intestines have structural and functional similarities with the intestines of mammals [55]. Flies and mammals have similar signaling pathways, such as Toll and Toll-like receptors, as well as similar protective immune mechanisms [56]. Genes involved in immune responses, such as signaling, gene expression, metabolism, immune system communication with other organs and systems, tissue homeostasis, intestinal physiology, development and metabolism, are preserved in both *Drosophila* and vertebrates [57].

The fruit fly *Drosophila* is widely used as an in vivo model to study bacterial [58], viral [59] and even fungal infections [60]. The insect is used as a model in the study of host-microorganism relationships, especially in relation to residential microflora, such as *L. planatrum*, which are nowadays commonly used in the food and pharmaceutical industries. The effects of *L. plantarum* on the host physiology are relevant for both fundamental and applied investigations aimed at using these bacteria as probiotics. Studies of the interactions between *Lactobacteria* and *Drosophila* are interesting because physiological, especially neurophysiological, and reproduction processes can be modulated through probiotics [61,62]. To date, particular relationship between *D. melanogaster* and its symbiote *L. plantarum* have revealed various facets of micro-macro-organism interaction [63,64]. *Lactobacilli* have been found to promote larvae [65,66] and protein production [67,68], regulate host dietary behavior [69,70,71] and induce the formation of active oxygen species (AFC) with NADP oxidase [72], thereby protecting fruit fly cells and tissues from damaging agents [73]. Available data on *L. plantarum* and *D. melanogaster* indicate the complex and ambiguous character of bacterial-host interactions. However, their potential role in protecting the host organism, as well as maintaining its homeostasis while infected with pathogens, could hardly be overstated.

There are many studies of the effect of probiotics on the *Drosophila melanogaster* model. Thus, the authors of [74] evaluated the effect of a probiotic drug on the locomotor functions of flies and changes in the composition of their intestinal microbiota. The following studies show the beneficial effect of probiotic drugs against pathogenic microorganisms for the treatment of flies [75,76].

Here we show that the *L. plantarum* AG10 strain exhibits strong antagonistic activity against several opportunistic intestinal microorganisms, and thus is capable of suppressing the growth of *S. aureus* and *E. coli* in vitro during milk fermentation; in addition, it can reduce the negative effects of *S. aureus* and *E. coli* in vivo in the survival model of *Drosophila melanogaster* at the embryonic (larval) and pupal stages. The whole genome sequencing allowed the identification, with high probability, the putative antimicrobial peptide responsible for the above activity. The genome contains extremely low amounts of putative virulence factors indicating that *L. plantarum* AG10 represents a promising probiotic.

## 2. Materials and Methods

### 2.1. Strains and Growth Conditions

*Lactiplantibacillus* (*Lactobacillus*) *plantarum* AG10 isolated from silage provides a high milk acidification rate and exhibited potential probiotic properties [50]. As a reference, we used the *Lactiplantibacillus plantarum* 8PA3 strain, which is approved as a probiotic strain (Biomed, Russia) and is widely used in various probiotic products, including “Lactobacterin”, “Biovestin-lacto”, “Lactonorm” and “Florin-forte”. It is also known for its effectiveness against diarrhea of various etiologies, dysbiosis, bacterial vaginosis, acute intestinal infection in children, as well as in the complex therapy of diseases associated with *Helicobacter pylori* [77].

LABs were stored in de Man, Rogosa and Sharpe (MRS) broth with 50% glycerol at −80 °C. Bacteria were seeded from the stock into MRS broth with the addition of 2% glucose and grown at 37 °C for 24 h. The obtained culture was added to skimmed milk to receive a starter culture.

*Escherichia coli* MG1655 (K-12), *Staphylococcus aureus* subsp. *aureus* ATCC 29213, *Klebsiella pneumonia* (clinical isolate), *Pseudomonas aeruginosa* ATCC 27853, *Serratia marcescens* (clinical isolate) and *Bacillus cereus* (clinical isolate) were used in this study as test bacteria for the evaluation of the antibacterial activity of the LABs. Clinical isolates of *B. cereus* and *K. pneumonia* were kindly provided by the Kazan Institute of Epidemiology and Microbiology (Kazan, Russia). The *S. marcescens* strain was kindly provided by the Institute of Medical Microbiology (Giessen, Germany).

*E. coli* and *S. aureus* strains carrying pCtuf Amp Gfpr plasmid providing constitutive GFP synthesis were used as markers of the presence of bacteria in the flies’ gut. The pCtuf Amp Gfpr plasmid was kindly provided by Prof. F. Götz, University Tübingen, Germany.

### 2.2. Antibacterial Activity of LAB Strains (Agar Drop Diffusion Test)

The overnight culture of *Lactobacilli* was inoculated as a lawn on MRS agar and incubated for 48 h at 37 °C. The agar blocks with *Lactobacilli* colonies were cut out with a sterile cork borer and set in Petri dishes onto a surface of solid LB medium inoculated with an 8- to 10-h culture of test organisms. The antagonistic activity was assessed after 24-h incubation at 37 °C by the diameter of growth inhibition zones of the test microorganisms around the agar blocks with *Lactobacilli* [78].

### 2.3. Antibacterial Activity of LAB Strains in the Fermented Milk

The antagonistic properties of LAB strains against pathogens during milk fermentation were tested after 1, 3, 7 and 21 days. *L. plantarum* 8PA3 was used as a reference strain. To obtain an LAB starter culture, an overnight culture of *Lactobacilli* was added to 5 mL of milk and incubated for 12 h at 37 °C. To 19 mL of milk, 1 mL of starter LAB culture (10^8^–10^9^ CFU/mL) was added; an overnight culture of *E. coli* or *S. aureus* were added to the final concentration of 10^6^ CFU/mL when desired and incubation was conducted for 8 h at 40 °C. Then, the samples were stored at 4 °C for 21 days. CFUs were counted using the drop-plate assay [79] with modifications [80,81]. Ten-fold dilution series of the fermented milk were prepared and plated drops (5 μL each) on differential media. MRS agar, mannitol-salt agar and Endo agar were used to differentiate LAB, *S. aureus* and *E. coli*, respectively. CFUs were counted from the two last drops, typically containing 5–15 colonies.

### 2.4. D. melanogaster Husbandry

All experiments were performed with virgin female *D. melanogaster*, since non-virgin females would have a chance to produce offspring from undesirable males. In the SMART test, the virgin females should be used to ensure the genotype clearance. Canton-*S*flies were used as wild-type strains in all following experiments unless otherwise specified. For the SMART assay, two mutant *Drosophila* lines were used: *mwh*, *flr^3^*, carriers of the multiple wing hairs (*mwh*, 3-0.3) and *flare^3^* (*flr^3^*, 3–38.8) marker genes. These lines were kept in thermostat stock in ¼ vol flasks containing culture medium for *D. melanogaster* (1000 mL water, 20 g *Saccharomyces cerevisiae* yeast, 7 g agar, 35 g sugar, 40 g semolina and 1 mL propionic acid) at 25 °C and 65% relative humidity.

Infection of flies with *L. plantarum* 8PA3, *L. plantarum* AG10, *E. coli* and *S. aureus* strains, as well as recombinant strains *E. coli* pCtufAmpGfpr and *S. aureus* pCtuf-gfpCmr with constitutive GFP synthesis, were performed through a nutrient substrate. For that, synchronous embryo clutches were obtained and transferred to the surface of the *Drosophila* nutrient medium with the addition of 100 μL of bacterial cell suspension washed in phosphate-buffered saline (10^7^–10^8^ CFU/mL in sterile PBS).

### 2.5. The Analysis of Flies Viability on Embryonic and Metamorphosis Stages

The frequency of lethal mutations during embryogenesis was used as an indicator of the changes occurring in gametes of imagoes. The frequency of embryo lethality was determined as the fraction of the eggs that stopped their development at a certain stage out of the total number of the eggs laid. To perform the experiment, virgin imagoes from all control and experimental groups were separated according to their sex within the first day after eclosion and kept separately in vials with temporary culture medium until they reached a sexually mature age (three days). Then, males and females were put together for 12 h for mating. Subsequently, inseminated females were placed in Petri dishes (d = 10 cm) with temporary medium (5 individuals per dish) for 12 h to obtain eggs. In the next day, the eggs were counted using a stereoscopic microscope and then placed in a thermostat (t = 24 °C) for 48 h. The sample size was 10 Petri dishes for each experimental group. The embryo lethality level was calculated according to [82].

Viability at the pupa stage was estimated as a percentage of those that did not hatch at the end of the period of emergence of adults from puparia in the offspring of five parental pairs. At the same time, the preimaginal death was assessed by changes in the morphology of the pupa.

### 2.6. Fertility Analysis

For the fertility analysis, five newly emerged females and five males were placed in vials with 10 mL of standard medium (control) and medium containing bacteria of all experimental groups for the oviposition period of 7 days. The vials were kept in an incubator until progeny appeared. Then, parental insects were removed from the tube. The number of adult males and females was fixed. For each experimental group, 10 vials were analyzed [83].

### 2.7. DNA Comet Assay

To assess DNA damage in the gut of flies, an alkaline variant of the DNA comet assay was used; this allows for the determination of single-strand DNA breaks in cells [84]. For all experimental groups, the gut of third-in star *Drosophila* larvae was isolated and mechanically suspended in the Poel’s salt solution (15 mMNaCl, 6.4 mM NaH_2_PO_4_, 42 mMKCl, 7.9 mM CaCl_2_, 1.8 mM KHCO_3_, 20.8 mM MgSO_4_; pH 6.95). Enterocytes were embedded in 0.75% agarose on slides. To prepare each single slide, a total of 5 guts were used. The slides were placed in a lysis solution (2.5 M NaCl, 100 mM EDTA, 10 mMTris, 1% Triton X-100; pH 10) for 1 h at 4 °C. Then the slides were incubated in an alkaline electrophoresis buffer (0.3 M NaOH, 1 mM EDTA, pH 13) for 10 min followed by electrophoresis at 15 V/300 mA for 10 min at 4 °C. Slides were washed 3 times in 20 mMTris (pH 7.5) and 3 times in distilled water and then fixed in ethanol for 10 min. Fluorescence microscopy (Carl Zeiss Axio Imager M2, Oberkochen, Germany) was used to visualize and rank the DNA comets.

### 2.8. SMART Test

The genotoxicity of *L. plantarum* strains and strains of *E. coli* and *S. aureus* was assessed using standard cross versions of SMART on *D. melanogaster*. Females from the *flr^3^/In(3LR)TM3* line were mated with *mwh/mwh* males [85,86,87]. The lines were kindly provided by O. N. Antosyuk, Ural Federal University (UrFU, Ekaterinburg, Russia). The emergent adults were collected and preserved in 70% ethanol. The preserved fly wings were placed under a stereoscopic microscope using entomological pincers and the wing pairs were spread over codified slides. A Faure solution (30 g gum Arabic, 20 mL glycerol, 50 g chloral hydrate, and 50 mL distilled water) was used to fix the wings. After mounting, the slides remained on a warm plate (60 °C) for 1 h. The spots (single or twin) on the wings were counted by using a Carl Zeiss Observer 1.0 microscope (Carl Zeiss, Oberkochen, Germany) at 400× magnification.

### 2.9. DNA Extraction

A single colony of *L. plantarum* AG10 was grown in Man–Rogosa–Sharpe (MRS) Broth (SigmaAldrich, St. Louis, MO, USA) under microaerophilic conditions at 37 °C overnight. Then, bacterial cells were harvested by centrifugation at 10,000 rpm for 5 min and the genomic DNA was extracted using GeneJET Genomic DNA Purification Kit (ThermoFisher, Waltham, MA, USA) according to the manufacturer’s protocol. The quality and purity of DNA was checked by using 0.7% agarose gel electrophoresis and OD260/OD280 ratio on Nanodrop2000 systems.

### 2.10. Genome Sequencing and Assembling

The *L. plantarum* AG10 whole genome sequencing was performed using a high-throughput IlluminaMiSeq platform. For IlluminaMiSeq sequencing, DNA was sheared to fragments ranging between 300 and 500 bp using the Covaris S220 (Covaris, Woburn, MA, USA) The fragmented DNA sample was end-paired, dA-tailed and ligated to multiple adapters. The ligated products were purified and further enriched using PCR, and paired-end sequencing was performed by using IlluminaMiseq (Illumina, San Diego, CA, USA). The quality of sequence reads was assessed using FastQC (version 0.11.9) [88]; the genome was assembled using SPAdes 3.15.3 [89]. The raw data of complete genome sequence of *L. plantarum* AG10 are available in NCBI GenBank database as BioProject PRJNA953814.

### 2.11. Bioinformatic Analysis

The assembled genome was annotated using Prokka [90]. Distribution of genes into subsystem categories was performed using the RAST server [91].

Genomic and proteomic alignments were performed using a Blast Global Alignment tool [92]. In silico screening for antimicrobial peptides was performed using online algorithms for the analysis of antimicrobial activity: dbAMP (https://awi.cuhk.edu.cn/dbAMP/ampfinder.php, accessed on 10 April 2023) [93], CAMPR3 (http://www.camp3.bicnirrh.res.in/, accessed on 10 April 2023) [94]. The VFDB database (http://www.mgc.ac.cn/VFs/main.htm, accessed on 12 April 2023) and VRprofile 2.0 (https://tool-mml.sjtu.edu.cn/STEP/STEP_VR.html, accessed on 13 April 2023) were used to search for proteins associated with bacterial virulence.

### 2.12. Statistical Analysis

All experiments were performed in biological triplicates with three repeats in each unless otherwise specifically stated. Statistical data analysis was performed using one-factor analysis of variance (one-way ANOVA) statistical test with Holm–Sidak correction for multiple testing in GraphPad Prism version 6.0 for Windows (GraphPad Software). For each indicator, the arithmetic mean, its estimation error and standard deviation were calculated. For data with non-Gaussian distribution, a non-parametric one-way analysis of variance (Kruskal–Wallis) test has been performed and median values with interquartile ranges were shown.

## 3. Results

### 3.1. In Vitro Antagonistic Activity of L. plantarum AG10

The antagonistic activity of *L. plantarum* AG10 was tested by the agar block method against *Bacillus cereus*, *Escherichia coli* MG1655, *Staphylococcus aureus* ATCC 29213, *Klebsiella pneumonia*, *Serratia marcescens* and *Pseudomonas aeruginosa* ATCC 27853 (see Table 1). *L. plantarum* 8PA3 was used as control strain. *P. aeruginosa* revealed highest sensitivity to antimicrobial substances produced by *L. plantarum* AG10, while *K. pneumoniae* and *B. cereus* were almost insusceptible. *E. coli*, *S. aureus* and *S. marcescens* exhibited weak susceptibility. By contrast, *L. plantarum* 8PA3 demonstrated antagonistic activity against *P. aeruginosa* only.

Further, the ability of *L. plantarum* AG10 to repress *E. coli* and *S. aureus* was evaluated in the skim milk fermentation model followed by storage over 21 days. To this end, pathogens were inoculated together with a starter culture and milk contamination was monitored. A probiotic strain of *L. plantarum* 8PA3 was used as a relevant control. The milk was incubated for 20 h at 40 °C under static conditions and then stored at 4 °C for 21 days. The number of viable cells of both lactobacilli and pathogenic microorganisms was evaluated by CFU counting after end of fermentation and after 3, 7 and 21 days of storage.

**Figure 1:**
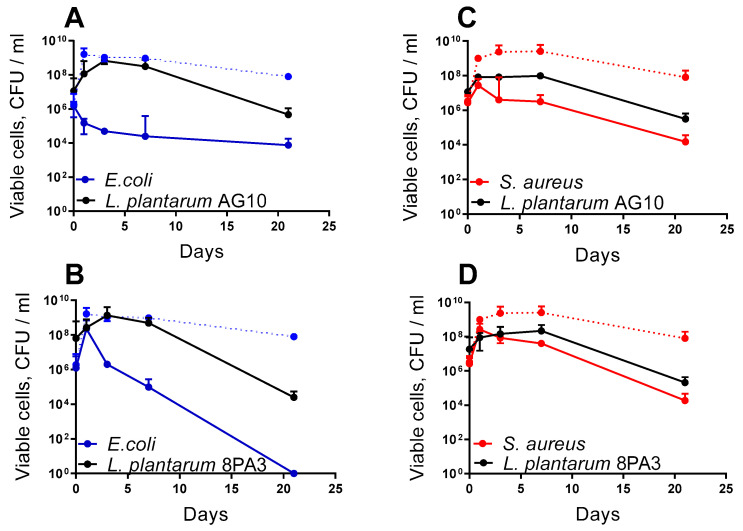
Antimicrobial activity against *E. coli* (**A**,**B**) and *S. aureus* (**C**,**D**)and repression of pathogenic bacteria by either *L. plantarum* AG10 (**A**,**C**) or *L. plantarum* 8PA3 (**B**,**D**) during co-cultivation in fermented milk and subsequent storage at a temperature of 4 degrees for 21 days with control points on 1, 3, 7 and 21 days by the CFU counting method. Median values with interquartile ranges are shown.

Overall, on the 21st day of storage, the amount of *L. plantarum* AG10 was slightly higher when compared to *L. plantarum* 8PA3, suggesting increased survival potential of the strain. In the presence of *L. plantarum* 8PA3, the viable *E. coli* completely disappeared up to the 21st day of the storage (Figure 1B). By contrast, while no complete death of the pathogen could be observed in the presence of *L. plantarum* AG10, the growth of *E. coli* was suppressed even during fermentation time, since the amount of CFUs was 10^5^ at the storage start (Figure 1A). Similarly, the number of viable *S. aureus* in the presence of *L. plantarum* AG10 was reduced 10-fold when compared to the *L. plantarum* 8PA3 co-culture, suggesting that *L. plantarum* AG10 is able to repress the growth of other bacteria during the fermentation period. During storage, CFUs of *S. aureus* continuously decreased in the co-culture of both *Lactobacilli* strains, with no significant differences in their growth repression rates.

### 3.2. Antagonistic Activity of Lactobacillus in the Drosophila Model

Tthe ability of *L. plantarum* strains to repress the growth of *S. aureus* and *E. coli* in vivo was evaluated on flies infected per-oral with these pathogens. Synchronous embryo clutches were transferred onto *Drosophila* nutrient medium; 100 μL of either *S. aureus* or *E. coli* suspension (10^7^–10^8^ CFU/mL in sterile PBS) were added as this concentration of viable pathogens has been used in some similar investigations [95,96,97,98]. Then, viability and reproduction were evaluated by counting the number of eggs laid, embryonic death, pupa death and fertility (number of the imagoes).

**Figure 2:**
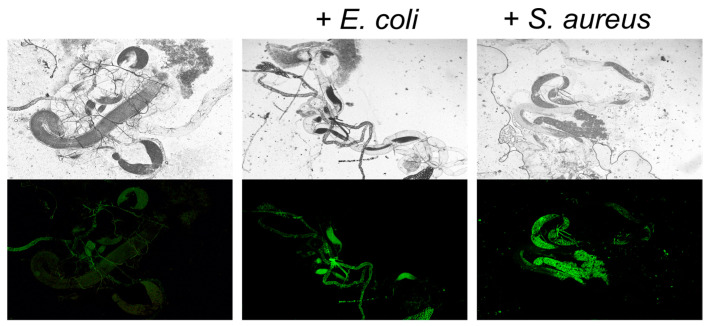
Fluorescence microscopy of gut of third stage *D. melanogaster* larvae of *L. plantarum* strains developed on media with either *E. coli* or *S. aureus*. Representative images of gut samples taken from five larvae in each group in three independent experiments are shown.

To confirm the uptake of *S. aureus* and *E. coli* by insects, the *E. coli* and *S. aureus* strains carrying pCtuf Amp Gfpr plasmid providing constitutive GFP synthesis were added to the growth medium with larvae. During the third stage of development, the larvaes’ gut was extracted and analyzed using fluorescence microscopy. As can be seen in Figure 2, strong fluorescence can be observed in the guts of larvae grown in medium with the addition of *E. coli* and *S. aureus* strains carrying pCtuf Amp Gfpr plasmid, suggesting the uptake of bacteria by larvae.

**Figure 3:**
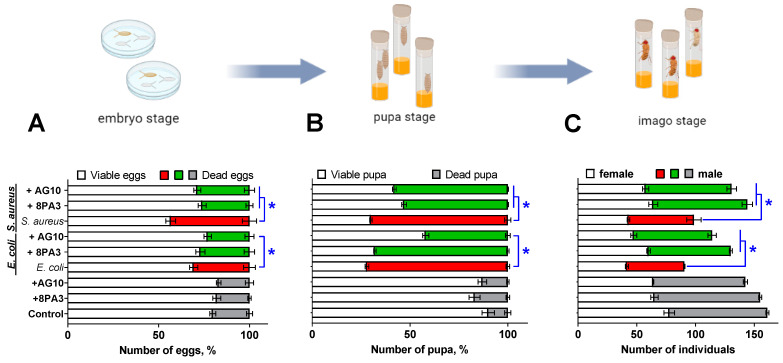
Antagonistic effects of *L. plantarum* strains against pathogens (*E. coli* and *S. aureus*) at embryo development (**A**), pupa morphogenesis (**B**) and fertility (**C**) stages of *D. melanogaster* development. Insects were grown on medium either without or with solely *Lactobacilli* (greys), solely pathogens (reds) or pathogens with *Lactobacilli* (greens) as indicated on the left. Asterisks indicate statistically significant differences (*p* < 0.05) determined using one-factor analysis of variance (one-way ANOVA) statistical test with Holm-Sidak correction for multiple testing between flies grown either in the presence of solely pathogen or with additional presence of *L. plantarum* strain as indicated (*n* = 10).

Neither the embryonic stage nor further development of the fruit flies was affected by the presence of *L. plantarum* 8PA3 and *L. plantarum* AG10 in the growth medium (Figure 3A greys). By contrast, the addition of either *E. coli* or *S. aureus* to the medium increased the frequency of embryonic death 1.5-fold (*p* < 0.05) and 2.2-fold (*p* < 0.05), respectively (Figure 3A reds). The simultaneous introduction of *Lactobacilli* with pathogens to the medium partially restored the survival of eggs (Figure 3A greens). A significant effect of the *L. plantarum* AG10 strain was observed, with the reduction of the embryonic death rate by 7% (*p* < 0.05) when co-cultured with *E. coli* and by 15%, (*p* < 0.05) when co-cultured with *S. aureus.* In the case of *L. plantarum* 8PA3, a significant effect could be observed only when co-cultured with *S. aureus* cells (reduction of embryonic death by 17%, *p* < 0.05).

Similar effects of *E. coli* and *S. aureus* on *Drosophila* viability, as well as its recovery following the addition of *L. plantarum* strains, have been observed at the pupa stage (Figure 3B). The presence of either *E. coli* or *S. aureus* in the medium increased the mortality rate of insects in the pupal stage by 60–62% when compared to the control (*p* < 0.05). However, in the presence of *L. plantarum* AG10, the mortality at the pupal stage was reliably reduced by 30% when compared to the group with solely *E. coli*, and by 12% when compared to the group with solely *Staphylococci* (*p* < 0.05). *L. plantarum* 8PA3 was able to reduce the mortality rate at the pupa stage by 17% (*p* < 0.05) but only in the group infected with *E. coli*.

Finally, Figure 3C exemplifies the effect of bacteria on the viability of insects at the imago stage. The number of viable individuals was reduced by 45% and 40% in the groups infected with either *E. coli* or *S. aureus* when compared to the control group, respectively. Similarly, while no significant effect of either of the *L. plantarum* strains could be observed when added solely to the flies’ growth medium, their presence could level out the negative effect of both pathogens, providing roughly similar amount of adults as in non-treated group.

### 3.3. Genotoxicity of Lactobacillus Strains in the Drosophila Model

**Figure 4:**
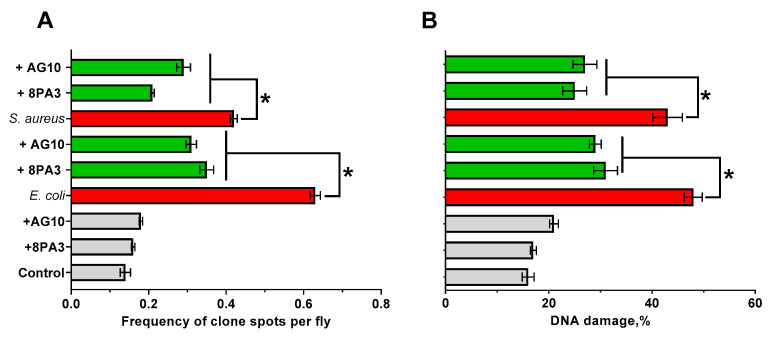
Suppression of *E. coli* and *S. aureus* genotoxicity by *L. plantarum* strains against *D. melanogaster.* (**A**) frequency of spots (SMART-test), (**B**) DNA damage index (DNA comet test). Insects were grown on medium either without or with solely *Lactobacilli* (greys), solely pathogens (reds) or pathogens with *Lactobacilli* (greens), as indicated on the left. Asterisks show statistically significant differences (*p* < 0.05) determined using one-factor analysis of variance (one-way ANOVA) statistical test with Holm–Sidak correction for multiple testing between flies grown in presence of solely pathogen and in addition of *L. plantarum* strain as indicated ((**A**) *n* = 10, (**B**) *n* = 30–50).

The potential genotoxicity of the strains [99] was evaluated by counting (i) the number of recombinant spots in the SMART test (definition of mutational/recombinant events) and (ii) the number of enterocytes of flies with DNA damage and the index of DNA comets (IDC, determination of damage in DNA). As can be seen in Figure 4, the *L. plantarum* strains alone did not demonstrate reliable genotoxic effect on the insects, while cultivation of flies in the presence of *E. coli* and *S. aureus* led to significant genotoxic effects (3–4 fold increase of spots frequency in the SMART test and DNA damage rate). The addition of either *L. plantarum* 8PA3 or *L. plantarum* AG10 together with the pathogens reduced the frequency of mutation and recombination events 1.5–2-fold.

### 3.4. Whole Genome Sequence of L. plantarum AG10

The whole genome of *L. plantarum* AG10 was sequenced using the high-throughput IlluminaMiSeq platform with overall coverage of 79× and sequence reads quality of 98.1% (probability of incorrect base call 0.1%). The *L. plantarum* AG10 genome consists of 109 contigs with the total length of 3,479,919 bp and 44.5% G + C content. The NCBI Bioproject has been deposited at NCBI under the accession number PRJNA953814 (https://www.ncbi.nlm.nih.gov/bioproject/PRJNA953814/, accessed on 8 April 2023) and consists of annotated genome and raw sequence data.

The genome annotation predicted 3467 coding sequences. Among them, 2129 open reading frames encode hypothetical proteins with unknown function; 1338 coding sequences encode proteins with known or predicted functions, 1222 of which can be distributed into 27 general metabolic groups (Table 2). The largest number of genes belongs to the carbohydrates (262 genes), the metabolism of amino acids and their derivatives (188 genes) and proteins (127 genes).

### 3.5. In Silico Identification of Antimicrobial Peptides and Virulence Factors in LAB Genomes

It was previously reported that LAB strains produce various antimicrobial peptides as antagonistic tools [31]. To evaluate whether the antagonistic properties of *L. plantarum* AG10 are associated with the production of bacteriocins or bacteriocin-like peptides, the whole genome of *L. plantarum* AG10 was analyzed in order to search for linear ribosomal peptides by using the AMP finder algorithm on the dbAMP server. Finally, three peptides (LLCBMPMO_01958, LLCBMPMO_01967 and LLCBMPMO_01968) with identity higher than 90% with peptides characterized by predicted antimicrobial potential were found (Table 3). In the *L. plantarum* 8PA3 genome, four peptides (WP_003641985.1, WP_003643800.1, WP_003643811.1 and WP_046947768.1) with high identity with antimicrobial peptides were found (Table 4). Further evaluation of antimicrobial properties of these peptides using CAMPR3 server indicated that the protein LLCBMPMO_01968 has the highest probability to exhibit antimicrobial properties (see Table 5). WP_003641985.1 has the highest probability of exhibiting antimicrobial properties according to the CAMPR3 server, while active only against *S. aureus* (see Table 5). WP_046947768.1 has lower scores in CAMPR3 but is predicted as active by DBAASP server. Thus, the activity of these peptides should be independently validated using an artificially synthesized peptide; this is planned as a part of our future work.

Further analysis of the *L. plantarum* AG10 genome using the VFDB database revealed, in silico, only three putative virulence factors (Table 6), while 30 were predicted for *L. plantarum* 8PA3 [100]; this apparently suggests the safety of *L. plantarum* AG10 as a probiotic strain.

## 4. Discussion

The widespread emergence of pathogenic bacteria resistant to conventional antimicrobials represents one of the key health challenges worldwide, and thus active screening of new approaches for the prevention and treatment of diseases is essential. Probiotic bacteria, such as lactic acid bacteria, *Bacilli* and their metabolites, have been intensively investigated as promising natural and eco-friendly substitutes to antibiotics [18,19,20,25,26].

Recent data indicate that the *L. plantarum* AG10 strain, isolated previously from clover silage, demonstrates promising antagonistic activity and probiotic properties in vitro [50,52]. In this work, we also show that it competes with pathogens in vivo, as well as diminishes their negative effects on the host. Besides the pronounced repression of most foodborne pathogens in the classic agar drop diffusion test (Table 1), this bacterium was able to suppress the growth of *S. aureus* and *E. coli* during milk fermentation. Thus, the amount of viable *E. coli* cells in the final product decreased from 10^6^ to 10^5^ after 8 h of milk fermentation by *L. plantarum* AG10 (Figure 1A); in the milk fermented by approved probiotic *L. plantarum* 8PA3, the viable *E. coli* cells increased until 10^8^ (Figure 1B). Similar suppression has been observed for *S. aureus* under the same conditions (Compare Figure 1C,D), suggesting antimicrobial potential of the strain. In addition, low amounts of putative virulence factors have been detected in silico in the genome of *L. plantarum* AG10 by using the VFDB database (in contrast to 30 found in *L. plantarum* 8PA3 [100]), suggesting its prominent safety profile as a potential probiotic.

The ability of *L. plantarum* AG10 to compete with pathogenic flora has been further demonstrated in vivo using a *Drosophila melanogaster* model. AG10 could diminish the negative effects of both *S. aureus* and *E. coli* on the survival characteristics of *Drosophila melanogaster* at both embryonic (larvae) and pupa stages, while the reference strain was active mainly against *S. aureus* only. Thus, the addition of either *L. plantarum* AG10 or the reference *L. plantarum* 8PA3 strain significantly reduced the mortality of *Drosophila* embryo and pupa infected with *S. aureus*, while *L. plantarum* AG10 could also level out the effects of *E. coli* on the survival of insects (Figure 3A,B). Consequently, in groups infected with pathogens in the presence of *Lactobacilli*, the amount of imago was similar to the untreated group or groups treated solely with *Lactobacilli* (Figure 3). Since bacteria added to the flies’ growth media were preliminary washed by PBS, the observed effects are apparently driven by antimicrobial substances produced by *Lactobacilli de novo.* Moreover, the experiment with *S. aureus* and *E. coli* overexpressing GFP confirmed that pathogens are captured by larvae (Figure 2), whereas successful introduction of *Lactobacilli* to the larvaes’ gut was shown previously [100], suggesting that the repression of pathogens occurs in vivo, while ex vivo interactions remain also possible.

The exact mechanism of the observed antimicrobial activity remains open to discussion. Lactic acid bacteria (LAB) compete with other bacteria by using various mechanisms such as synthesis of organic acids (mainly lactic acid) and acidification of the broth [31], hydrogen peroxide production [32] and secretion of antimicrobial peptides, as well as bacteriocins [33,34]. While broth acidification seems to be the main factor of antagonism in both agar drop diffusion and milk fermentation tests, antimicrobial peptides could play the leading role in vivo [33,34]. Moreover, it has been reported previously that *L. plantarum* AG10 acidifies the milk in a less pronounced manner than the industrial strains [52,53], assuming that the production of antimicrobial peptides could be apparently responsible for the observed effects both in vitro and in vivo. Indeed, the screening of putative antimicrobial peptides in the genome of *L. plantarum* AG10 in comparison with the dbAMP database allowed identification of three peptides with over 90% homology with peptides characterized by presumable antimicrobial activity (Table 3). Among them, LLCBMPMO_01967 seems to be active only against *S. aureus*, while LLCBMPMO_01968 exhibit antimicrobial properties against both *S. aureus* and *E. coli* with a high probability (Table 5). These findings agree with observations of the apparent repression of pathogenic bacteria in *Drosophila* (Figure 3 and Figure 4). The reference strain *L. plantarum* 8PA3 also carries four putative AMPs in the genome (Table 4) with WP_046947768.1, with predicted activity against both pathogens for the latter (Table 5). Nevertheless, during in vivo experiments, *L. plantarum* 8PA3 did not reduce the mortality in *E. coli* infected group (Figure 3 and Figure 4), suggesting that this AMP is apparently either inactive or non-expressed under experimental conditions. Thus, despite the relatively high possibility of antimicrobial properties predicted for AMPs in silico (Table 4), an independent in vitro verification of their activity is required in further investigations.

## 5. Conclusions

Taken together, these data allow the suggestion that the *L. plantarum* AG10 strain is a promising candidate for use in dairy production, to suppress the growth of pathogens during milk fermentation, and in livestock food supplements, as a preservative from foodborne infections. While our data demonstrated that the *L. plantarum* AG10 strain is capable of reducing the negative effects of *S. aureus* and *E. coli* in vivo in the survival model of *Drosophila melanogaster* at the embryonic (larval) and pupal stages, multiple additional tests are required to validate the safety and efficiency of *lactobacilli* as a natural and eco-friendly alternative to antimicrobials. We plan to examine this in our future research. The whole genome sequencing allowed identification, with high probability, of the putative antimicrobial peptide responsible for the above activity. The genome contains extremely low amounts of putative virulence factors, indicating that *L. plantarum* AG10 represents a promising probiotic.

## Figures and Tables

**Table 1:** Antimicrobial activity of LAB strains evaluated by agar block method (inhibition zones, mm). Data are present as averages ± SD from three biological triplicates with three repeats in each.

LAB Strain	Growth Inhibition, mm					
	*B. cereus*	*E. coli*	*S. aureus*	*K. pneumoniae*	*S. marcescens*	*P. aeruginosa*
*L. plantarum* AG10	1.0 ± 0.09	2.3 ± 0.20	2.8 ± 0.24	1.0 ± 0.70	3.0 ± 0.24	5.0 ± 0.36
*L. plantarum* 8PA3	1.0 ± 0.11	1.5 ± 0.13	1.5 ± 0.13	1.0 ± 0.80	1.5 ± 0.60	2.5 ± 0.14

**Table 2:** The genes distribution over 27 general COG functional categories.

Number of Genes	Description
262	Carbohydrates
188	Amino acids and derivatives
127	Protein Metabolism
112	Cofactors, vitamins, prosthetic groups, pigments
90	Nucleosides and nucleotides
77	Cell wall and capsule
65	DNA metabolism
41	RNA metabolism
39	Virulence, disease and defense
38	Fatty acids, lipids, and isoprenoids
37	Membrane transport
30	Phages, prophages, transposable elements, plasmids
21	Stress response
16	Regulation and cell signaling
15	Respiration
14	Miscellaneous
9	Nitrogen metabolism
8	Metabolism of aromatic compounds
6	Dormancy and sporulation
6	Phosphorus metabolism
5	Potassium metabolism
5	Iron acquisition and metabolism
4	Cell division and cell cycle
4	Secondary metabolism
3	Sulfur metabolism
0	Photosynthesis
0	Motility and chemotaxis

**Table 3:** Putative antimicrobial peptides of *L. plantarum* AG10 genome detected in silico with the dbAMP server.

Protein ID	*L. plantarum* AG10 Protein Sequence	AMP ID	Identity, %	Alignment Length
LLCBMPMO_01958	MNKMIKNLDVVDASAPISNNKLNGVVGGDAWKNFWSSLRKGFYDGEAGRANPSLINGLKLRRAYSGNQINY	dbAMP_09551	92	50
		dbAMP_02440	100	21
LLCBMPMO_01967	MKKFLVLRDRELNAISGGVFHAYSARGVRNNYKSAVGPADWVISAVRGFIHG	dbAMP_11717	100	34
LLCBMPMO_01968	MLQFEKLQYSRLPQKKLAKISGGFNRGGYNFGKSVRHVVDAIGSVAGIRGILKSIR	dbAMP_08433	100	56
		dbAMP_02258	100	33
		dbAMP_02257	91	33

**Table 4:** Putative antimicrobial peptides of *L. plantarum* 8PA3 genome detected in silico with the dbAMP server.

Protein ID	*L. plantarum* 8PA3 Protein Sequence	AMP ID	Identity	Alignment Length
WP_003641985.1	MLQFEKLQYSRLPQKKLAKISGGFNRGGYNFGKSVRHVVDAIGSVAGIRGILKSIR	dbAMP_08433	100	56
		dbAMP_02258	100	33
		dbAMP_02257	91	33
WP_003643800.1	MDKFEKISTSNLEKISGGDLTTKLWSSWGYYLGKKARWNLKHPYVQF	dbAMP_06066	100	28
WP_003643811.1	MKKFLVLRDRELNAISGGVFHAYSARGVRNNYKSAVGPADWVISAVRGFIHG	dbAMP_11717	100	34
WP_046947768.1	MKIQIKSMKQLSNKEMQKIVGGKSSAYSLQMGATAIKQVKKLFKKWGW	dbAMP_07932	98	48
		dbAMP_05363	100	26
		dbAMP_00773	100	23
		dbAMP_12287	100	22
		dbAMP_12286	95	21
		dbAMP_02438	100	17

**Table 5:** Evaluation of antimicrobial properties for *L. plantarum* AG10 and *L. plantarum* 8PA3 peptides with the CAMPR3 and DBAASP servers.

N	The Protein Precursor	CAMPR3			DBAASP	
		SVM	Random Forest	DAC	*E. coli* ATCC 25922	*S. aureus* ATCC 25923
1	LLCBMPMO_01958	0.399	0.556	0.399	Not Active (0.70)	Not Active (0.78)
2	LLCBMPMO_01967	0.230	0.347Koheцфoрмы	0.230	Active (0.51)	Not Active (0.61)
3	LLCBMPMO_01968	0.757	0.812	0.757	Active (0.53)	Active (0.50)
4	WP_003641985.1	0.716	0.757	0.812	Active (0.54)	Not Active (0.51)
5	WP_003643800.1	0.096	0.122	0.023	Not Active (0.63)	Not Active (0.69)
6	WP_003643811.1	0.189	0.230	0.347	Not Active (0.60)	Not Active (0.62)
7	WP_046947768.1	0.477	0.610	0.500	Active (0.74)	Active (0.60)

**Table 6:** Virulence factors detected in silico in *L. plantarum* AG10 genome (based on the VFDB database).

*L. plantarum* AG10 Protein	Gene Locus	Bacterial Virulence Factor
ATP-dependent Clp endopeptidase proteolytic subunit ClpP	QBL19_01375	ATP-dependent Clp protease proteolytic subunit (*Listeria*)
UTP-glucose-1-phosphate uridylyltransferase GalU	QBL19_01520	UTP-glucose-1-phosphate uridylyltransferase HasC (product);
		Hyaluronic acid capsule (relative VF)
		(*Streptococcus*)
choloylglycine hydrolase	QBL19_03780	Bile salt hydrolase (*Listeria*)

## Data Availability

All data are available in manuscript.
